# Isolation of extra-cellular vesicles in the context of pancreatic adenocarcinomas: Addition of one stringent filtration step improves recovery of specific microRNAs

**DOI:** 10.1371/journal.pone.0259563

**Published:** 2021-11-16

**Authors:** Yi-Fan Xu, Xiaohui Xu, Kritisha Bhandari, Amy Gin, Chinthalapally V. Rao, Katherine T. Morris, Bethany N. Hannafon, Wei-Qun Ding

**Affiliations:** 1 Department of Pathology, Stephenson Cancer Centre, College of Medicine, University of Oklahoma Health Sciences Center, Oklahoma City, Oklahoma, United States of America; 2 Department of General Surgery, First People’s Hospital of Taicang City, Taicang Affiliated Hospital of Soochow University, Suzhou, China; 3 Department of Medicine, Hematologic Oncology Section, Stephenson Cancer Centre, College of Medicine, University of Oklahoma Health Sciences Center, Oklahoma City, Oklahoma, United States of America; 4 Department of Surgery, Stephenson Cancer Centre, College of Medicine, University of Oklahoma Health Sciences Center, Oklahoma City, Oklahoma, United States of America; 5 Department of Obstetrics and Gynecology, Gynecologic Oncology Section, Stephenson Cancer Centre, College of Medicine, University of Oklahoma Health Sciences Center, Oklahoma City, Oklahoma, United States of America; Gustave Roussy, FRANCE

## Abstract

microRNAs (miRNA) in extracellular vesicles (EVs) have been investigated as potential biomarkers for pancreatic ductal adenocarcinoma (PDAC). However, a mixed population of EVs is often obtained using conventional exosome isolation methods for biomarker development. EVs are derived from different cellular processes and present in various sizes, therefore miRNA expression among them is undoubtedly different. We developed a simple protocol utilizing sequential filtration and ultracentrifugation to separate PDAC EVs into three groups, one with an average diameter of more than 220 nm, named operational 3 (OP3); one with average diameters between 100–220 nm, named operational 2 (OP2); and another with average diameters around 100 nm, named operational 1 (OP1)). EVs were isolated from conditioned cell culture media and plasma of human PDAC xenograft mice and early stage PDAC patients, and verified by nanoparticle tracking, western blot, and electronic microscopy. We demonstrate that exosome specific markers are only enriched in the OP1 group. qRT-PCR analysis of miRNA expression in EVs from PDAC cells revealed that expression of miR-196a and miR-1246, two previously identified miRNAs highly enriched in PDAC cell-derived exosomes, is significantly elevated in the OP1 group relative to the other EV groups. This was confirmed using plasma EVs from PDAC xenograft mice and patients with localized PDAC. Our results indicate that OP1 can be utilized for the identification of circulating EV miRNA signatures as potential biomarkers for PDAC.

## Introduction

miRNA expression is deregulated in pancreatic ductal adenocarcinoma (PDAC) [1, 2] and circulating miRNAs have been explored for the purpose of developing new PDAC biomarkers [3–7]. However, no miRNA signatures have been established to screen for PDAC in the clinical setting. One major obstacle is that miRNAs isolated from serum or plasma are undoubtedly heterogeneous, originating from different cellular sources and presented in various forms, such as extracellular vesicle-encapsulated or protein-bound miRNAs. This inherent heterogeneity reduces the sensitivity and potential of circulating miRNAs as biomarkers for PDAC.

Several groups of extracellular vesicles (EVs) have been identified thus far, including microvesicles, exosomes, and apoptotic bodies, which differ in size, biogenesis, and biological function [8]. Among them, exosomes are EVs around 100nm in size and present in almost all biological fluids [9–11]. Cancer cells secrete more exosomes than normal cells, which are released into the tumor microenvironment and the circulation [12–17]. Therefore, exosome components collected from biological fluids may serve as biomarkers for PDAC [18–22]. We have recently demonstrated that plasma exosome miR-196a and miR-1246 are potential biomarkers for localized PDAC [22]. However, results from these previous reports, including ours, indicate that the detection of plasma exosome miRNAs is not sensitive or specific enough to serve as clinically applicable biomarkers for PDAC. New strategies are required to improve isolation methods and the specificity and sensitivity of detection in order to increase the potential of exosome miRNAs as diagnostic or prognostic indicators for early stage PDAC.

Conventional techniques for exosome isolation from biological fluids, such as ultracentrifugation or polymer-based commercial kits, cannot completely differentiate exosomes from other EVs, especially microvesicles, due to overlap in certain physical characteristics such as size and density [23]. Consequently, miRNA expression in isolated EVs reflects a mixed miRNA signal from both exosomes and microvesicles. Considering that different groups of EVs are derived from different subcellular origins [8], miRNA expression in exosomes highly likely differs from those in microvesicles. In this context, we have developed a new simple protocol to separate PDAC EVs into three groups based on their sizes; one group of EVs with an average diameter of approximately 100nm (OP1 group), one with an average diameter between 100-220nm (OP2 group), and those with an average diameter greater than 220nm (OP3 group). Expression of miR-1246 and miR-196a, both highly enriched in PDAC exosomes [22], was analyzed in the three groups of EVs. We found that exosomes are highly enriched in the OP1 group of EVs and expression of miR-196a is significantly higher in the OP1 group compared to the OP2 and OP3 groups derived from PDAC cells and patient plasma, supporting the concept that size-restricted isolation of EVs from patient plasma is a promising strategy to increase the sensitivity of selected EV miRNAs as biomarkers for PDAC.

## Materials and methods

### Cell culture

The human pancreatic cancer cell line PANC-1 and human pancreatic ductal epithelial cell line hTERT-HPNE were obtained from the American Type Culture Collection (ATCC, Manassas, VA, USA) and cultured according to ATCC’s instructions. Cell culture medium was supplemented with 10% exosome-depleted fetal bovine serum (FBS) and 1% penicillin-streptomycin. Exosome-depleted FBS was prepared by pelleting the serum exosomes at 100,000 × g for 2 h at 4°C, and the resulting supernatant was filtered through a 0.22μm filter. Cells were maintained in a humidified chamber at 37°C, 5% CO_2_, and passaged twice a week.

### Patient plasma samples

Plasma samples (n = 7, Table 1) were collected from patients with localized pancreatic cancer, who underwent primary tumor biopsy or resection at the Stephenson Cancer Center, University of Oklahoma Health Science Center (OUHSC). Blood was collected prior to biopsy or resection as described [22]. Control plasma samples (n = 7) were collected from donors with no history of pancreatic cancer (mean age = 58.3; 3 females and 4 males) from the Oklahoma Blood Institute, Oklahoma City, OK, USA. The study was approved by the OUHSC IRB committee (#5535 and #7565) and written informed consent was obtained from all participants. Plasma was separated by centrifugation and stored at -80°C. Patient information including age, tumor size, clinical stage and histological grade were obtained from the clinical and pathological records, and clinical stages were classified using the tumor, node, metastasis (TNM) system (AJCC 8^th^ edition).

**Table 1:** Clinicopathological features of PDAC patients included in the study.

Sample	Age	Sex	Tumor size	Stage^a^
PL1	49	male	2.5*2.8 cm	IIA
PL2	85	female	1.8*0.7*0.4 cm	IIA
PL3	70	male	0.6*0.4 cm	IIA
PL4	84	male	4.5 *2.5 cm	IIA
PL5	64	female	0.7*0.5 cm	I
PL6	60	female	UN	I
PL7	70	male	1.5*1.0 cm	IIA
Mean Age	68.85			
Median Age	70			

^a^ Pathologic TNM tumor staging, (American Joint Committee on Cancer Care 7^th^ Edition).

### Animal experiment

Five-week-old male NCr nude mice (NCRNU-M) were purchased from Taconic Farms Inc. (Germantown, NY) and used for this study. The research protocol was approved by the Institute Animal Care and Use Committee at OUHSC and in accordance with the Institute Animal Care and Use Committee procedures and guidelines. PANC-1 cells (2 x 10^6^) were suspended in 200μl PBS-Matrigel and injected into the flanks of mice subcutaneously. Three groups of mice with 6 mice per group were utilized. Group 1, without tumor cell inoculation; group 2, inoculated with PANC-1 and euthanized 1-week post inoculation; and Group 3, inoculated with PANC-1 and euthanized 2 weeks post inoculation (when tumor size reached to 5–8 mm in diameter). Tumor volume was calculated using the following formula: V = 1/2(A×B^2^), where V is the tumor volume, A is the length, and B is the width of the xenograft. The tumor volume and body weight were monitored 3 times a week. The blood was collected through cardiac puncture after mice being euthanized by CO_2_ asphyxiation (30% per min air displacement in the CO2 chamber) and plasma was separated through centrifugation [24]. This animal experiment was performed in 2017.

### Extracellular vesicle isolation

**Fig 1:**
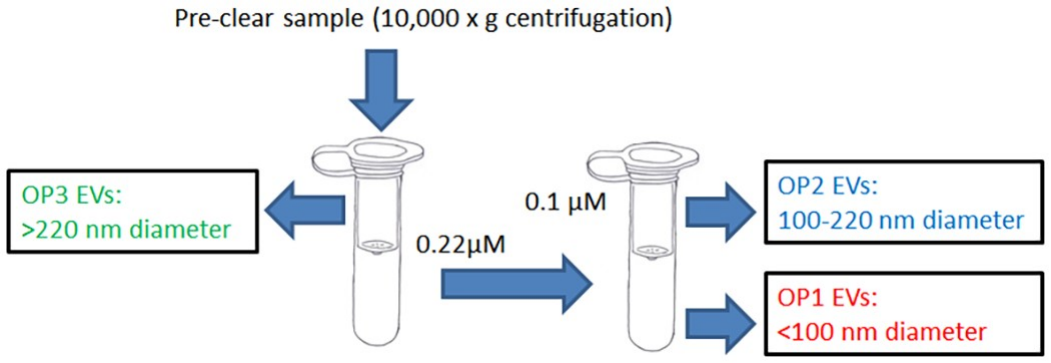
Isolation and separation of EVs by PVDF filtration and ultracentrifugation. The culture medium of PANC-1 cells was pre-cleared by 10,000g centrifugation for 30 min at 4°C, and the resulting supernatant was passed through a 0.22μm PVDF centrifuge filter. The operational 3 (OP3) EVs (>220 nm) were trapped in the first filter. The filtered supernatant was then applied to a 0.1μm PVDF centrifuge filter. The operational 2 (OP2) EVs (100–220 nm) were trapped in the second filter and re-suspended in PBS from the filter surface. The operational 3 (OP1) EVs (<100 nm) in the final supernatant were recovered by ultracentrifugation (100,000g, 70 min at 4°C). For plasma EV isolation and separation, we started with the isolation of EVs using the Exoquick reagent. The resulting EVs were dissolved in PBS and applied to the filtration and separation process as depicted above.

EVs of different sizes were isolated from the conditioned cell culture medium utilizing a combination of centrifugation, ultracentrifugation, and PDVF filters with different pore sizes as described in Fig 1. Plasma EVs were first isolated using the Exoquick reagent (System Biosciences, Mountain View, CA, USA) to gain higher yield following the manufacturer’s protocol and diluted in PBS. The EVs from patient plasmas were filtered twice and pelleted as shown in Fig 1. OP3 was obtained by a thorough washing of the 220nm filter after filtration with 100μl PBS; OP2 was collected via thoroughly washing the 100nm filter after filtration with 100μl PBS; and OP1 in the flow through were pelleted at 100,000g for 70 min 4°C. The separated EVs were verified by nanoparticle tracking analysis, western blot and electronic microscope.

### Western blot analysis

Protein lysates from the three groups of EVs (Fig 1) were prepared by resuspending them in RIPA Buffer (50mM Tris-HCl pH 7.4, 150mM NaCl, 0.5% sodium deoxycholate, 1% NP-40, and 0.1% sodium dodecyl sulfate) containing 1 mM phenlymethylsulfonyl fluoride, 5μg/ml leupeptin, 2μg/ml aprotinin, and 1μg/ml pepstatin A [24]. Approximately 40μg of protein from each sample was separated on a 10% SDS-PAGE gel (non-reducing gel for CD63), transferred to a polyvinylidene fluoride membrane, blotted with the following primary antibodies: CD63, CD81, TSG101 (Santa Cruz Bio Technology Inc., CA, USA), Flotillin-1 and Calnexin (Cell Signaling Technology, Inc., MA, USA), and secondary IgG-HRP (Santa Cruz Bio Technology Inc., CA, USA).

### Nanoparticle tracking analysis

Isolated EVs were diluted in PBS and analyzed using the Nanosight NS300 System (Malvern Instruments, UK) which is equipped with a blue laser (405 nm) [24]. Nanoparticles illuminated by the laser were captured for 60 s. The Nanosight Tracking Analysis software was used to provide size distribution profiles.

### Electron microscopy

EVs were examined under an electron microscope as we previously described [24]. In short, EVs were fixed in 2% paraformaldehyde and absorbed onto formvar coated copper grids. Samples were fixed in 1% glutaraldehyde and rinsed in distilled water. Samples were stained with uranyl oxalate followed by methyl cellulose uranyl acetate on ice. Imaging was acquired on a Hitachi H7600 microscope.

### RNA extraction

Total RNA was extracted from EV pellets using the TRIzol reagent, and further purified with the PureLink column following the manufacturer’s protocol^24^ (Invitrogen/ThermoFisher, Carlsbad, California). RNA concentration was quantitated using the NanoDrop ND-100 Spectrophotometer (NanoDrop Technologies, Wilmington, DE, USA).

### Quantitative real-time reverse transcription PCR

For miRNA expression analysis, complementary DNA from 80 ng of total RNA was synthesized using the qScript miRNA cDNA Synthesis Kit (Quanta BioSciences, Inc., Beverly, MA, USA). A cDNA aliquot, equivalent to 4 ng of the original RNA, was mixed with Perfecta SYBR Green SuperMix, Universal PCR Primer and specific forward primers for miR-1246 (5’-GCGCGATGGATTTTTGGAGCAG-3’, MIMAT0005898) or miR-196a-5p (5’-GCGTAGGTAGTTTCATGTTGTTGGG-3’, MIMAT0000226) in 20 μl qPCR reactions. A synthetic *Caenorhabditis elegans* miR-54 (cel-miR-54) RNA oligonucleotide (Integrated DNA Technologies, Coralville, IA, USA) was spiked-into RNA samples as an internal control^33^ (5’-UACCCGUAAUCUUCAUAAUCCGAG-3’). PCR reactions were run on a Bio-Rad CFX 96 Real-Time PCR (Bio-Rad, Hercules, CA, USA) instrument under following conditions: 95°C for 2 min, 40 cycles of 95°C for 5 s, 60°C for 15 s and 70°C for 15 s. Changes in gene expression were calculated using the ΔΔ*C*_*T*_ method as follows: Δ*C*_*T*_ = *C*_*T*_ (target miRNA) − *C*_*T*_ (cel-miR-54); ΔΔ*C*_*T*_ = Δ*C*_*T*_ (cancer EV miRNA)- Δ*C*_*T*_ (normal EV miRNA); the fold changes = 2^−ΔΔ*CT*^.

### Statistical analysis

Statistical analyses were performed using the GraphPad Prism software (GraphPad Software, Inc. La Jolla, CA, USA). Student’s t test was used to determine significant differences between control and experimental groups.

## Results

### Isolation and separation of EVs

**Fig 2:**
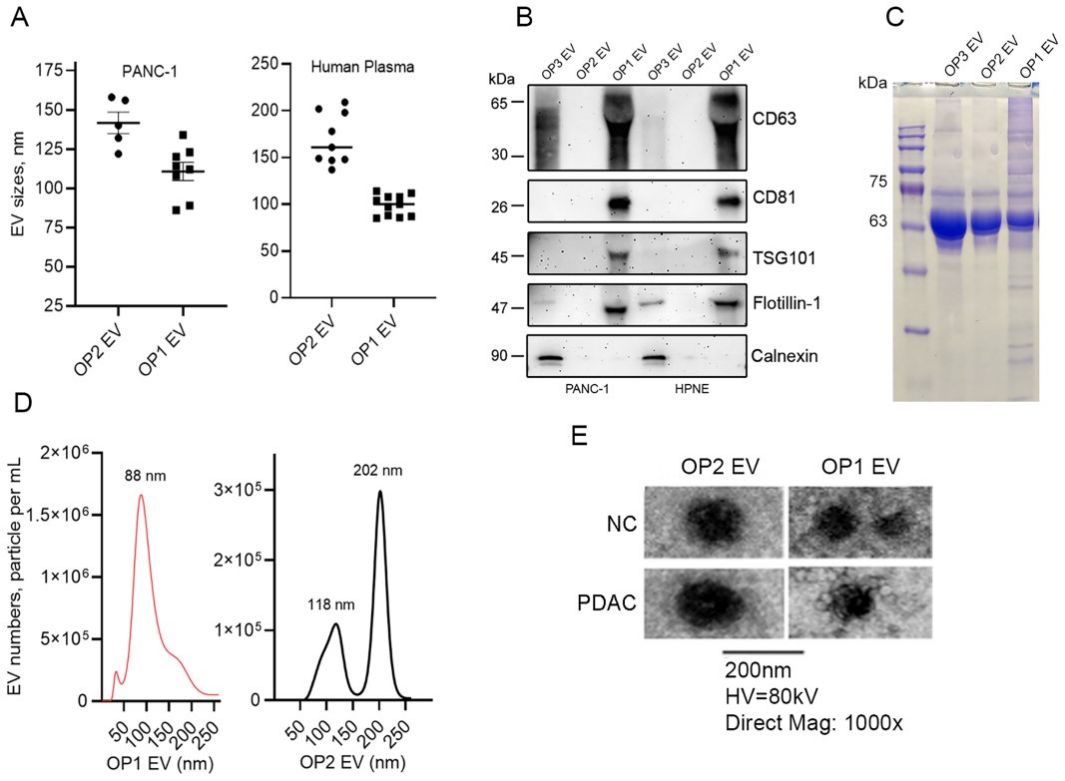
Characterization of EVs by nanoparticle tracking analysis, exosome marker detection, and electron microscopic examination. ***A***. Nanoparticle tracking analysis of OP1and OP2 EVs derived from PANC-1 cells and healthy donor human plasma. ***B***. Western blot analysis of CD63, CD81, TSG101, flotillin-1, and calnexin in the OP1, OP2, and OP3 EVs derived from PANC-1 cells (40μg protein each lane, non-reducing condition for CD63, n = 3). ***C***. Coomassie blue staining of the SDS-PAGE gel loaded with proteins from OP1, OP2 and OP3 EVs (25μg each lane). ***D***. Representative nanoparticle tracking analysis of OP1 and OP2 EV concentrations (PANC-1). ***E***. Representative electronic microscopic images of OP1 and OP2 EVs (Human plasma).

EVs produced by PANC-1 cells were isolated by filtration and ultracentrifugation (Fig 1). In brief, cell debris was pre-cleared by low speed centrifugation. We then applied the supernatant through a filter with a pore size of 0.22μm (220nm) and the EVs retained on the filter were larger than 0.22μm in diameter (OP3) and are likely a mixture of microvesicles and apoptotic bodies [8]. The EVs that passed through the 0.22μm filter were further separated into two groups using a second PVDF filter with a pore size of 0.1μm (100nm). A OP2 group with a diameter approximately 100 to 220nm in size was recovered by washing the filter and a OP1 group with a diameter less than or around 100nm in size was recovered from the flow-through. For the plasma samples, we initiated the EV collection beginning with the Exoquick reagent, and the precipitated EVs were dissolved in PBS. The sequential filtration process as shown in Fig 1 was then followed. The isolated EVs were verified by nanoparticle tracking analysis and western blot for detection of the well-established exosome surface markers CD63, CD81, TSG101, and flotillin. As shown in Fig 2, we successfully separated two groups of EVs, with significant differences in particle sizes in both cell line-derived and plasma-derived EVs (Fig 2A). Established exosome markers were detected primarily in the OP1 group, and not in the OP2 and OP3 group. A negative control calnexin was only detected in the OP3 group, and not the OP2 or OP1 group, indicating that exosomes are enriched in the OP1 group (Fig 2B). Coomassie blue staining confirmed the loading of proteins from each group of EVs onto the SDS-PAGE gel (Fig 2C). It seemed clear that the amount of protein and RNA from OP2 is proportionally lower than that from OP1 when both groups of EVs were isolated from the same amount of culture medium (S1 Table). Representative nanoparticle tracking analysis of PANC-1-derived EVs (Fig 2D) and electronic microscopic images of plasma-derived OP1 and OP2 EVs (Fig 2E) are also shown.

### miRNA expression in PDAC cell line-derived EVs

**Fig 3:**
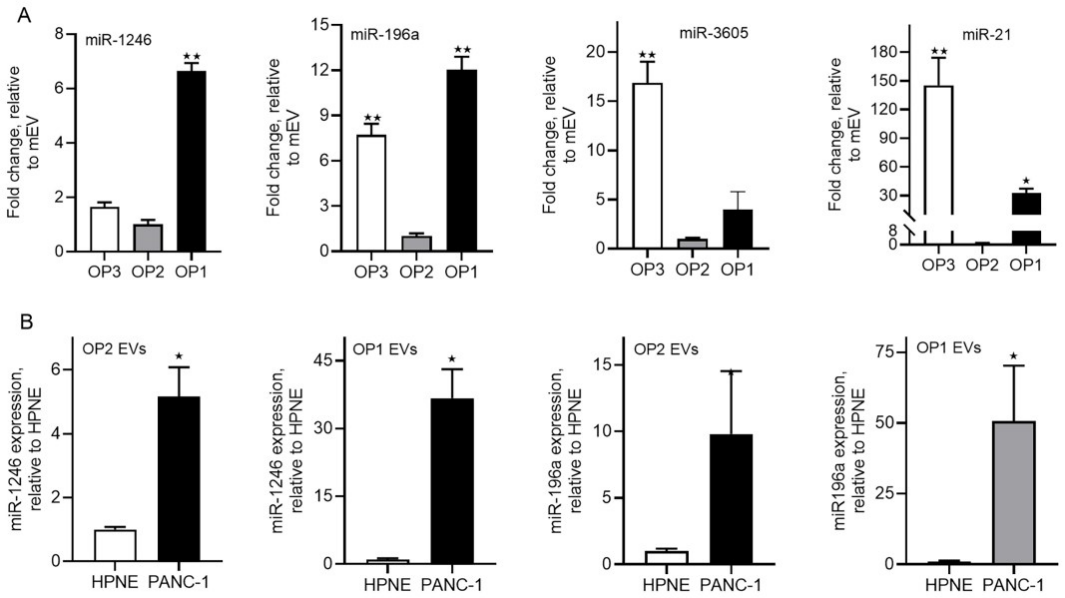
Expression of miR-1246 and miR-196a in the OP1, OP2 and OP3 groups of EVs derived from PANC-1 and hTERT-HPNE cells. ***A***. qRT-PCR (80ng total RNA used for each cDNA synthesis) analysis of miR-1246, miR-196a, miR-21, and miR-3605 expression in the OP1, OP2 and OP3 group of EVs derived from PANC-1 cells. ***B***. A comparison of miR-1246 and miR-196a expression between PANC-1- and hTERT-HPNE-derived OP1 and OP2 group of EVs. *. P<0.05, **, p<0.01, unpaired Student t-test (n = 3–4).

We have recently reported that miR-1246 and miR-196a are selectively enriched in PDAC cell line-derived exosomes and that plasma exosome miR-196a is a potential biomarker for the detection of early stage PDAC [22]. However, in that study exosomes were isolated by ultracentrifugation combined with filtration using a 0.22μm filter, which could not separate the OP1 and OP2 groups. We therefore analyzed expression of these two miRNAs, along with expression of miR-21 and miR-3605, in different groups of EVs isolated from PANC-1 and hTERT-HPNE cells. Interestingly, the expression of miR-1246 and miR-196a was significantly higher in the OP1 group compared to the OP2 and OP3 groups, whereas expression of miR-21 and miR-3605 was significantly higher in the OP3 group (Fig 3A). Moreover, the expression of miR-1246 and miR-196a in EVs derived from PANC-1 cells was significantly higher than in EVs derived from HPNE cells (normal pancreatic ductal epithelial cell) and with greater magnitude in the OP1 group relative to the OP2 group (Fig 3B).

### miRNA expression in plasma EVs from PANC-1 xenografted mice

**Fig 4:**
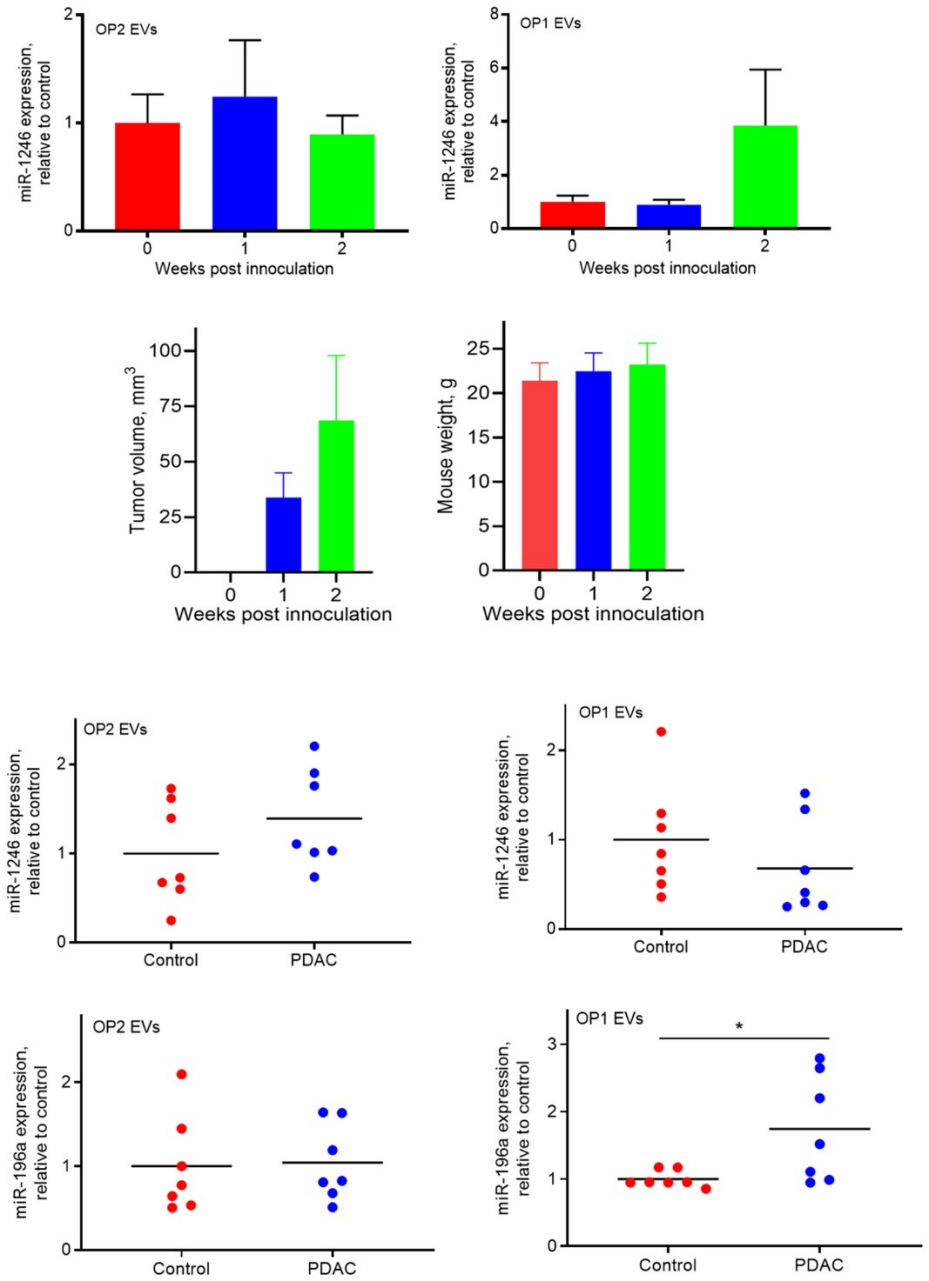
Expression of miR-1246 in OP1 and OP2 groups of EVs derived from the plasma of xenografted nude mice and PDAC patients/healthy controls. Plasma were collected from xenografted mice at 1 and 2 weeks post PANC-1 inoculation, and from patients with early stage PDAC and healthy controls. ***Top***. qRT-PCR (80ng total RNA used for each cDNA synthesis) analysis of miR-1246 expression in the OP2 (***left***) and OP1 (***right***) group of EVs derived from the plasma of xenografted nude mice and control mice (n = 6). ***Middle***. Tumor volume (***left***) and mice weight (***right***) are shown. ***Low***. qRT-PCR (80ng total RNA used for each cDNA synthesis) analysis of miR-1246 expression (***top panel***) and miR-196a expression (***low panel***) in the OP1 and OP2 group of plasma EVs between PDAC patients and healthy donors (control). *, p<0.05, unpaired Student t-test (n = 7). Individual values of detection are presented in the supplement material.

We have recently demonstrated that plasma exosome miR-1246 is elevated in mice carrying human xenograft tumors [24]. Because there is no mouse version of miR-1246 (miRBase.org) and miR-1246 is the most enriched miRNA in PANC-1 cell-derived exosomes [22], to determine whether the size of EVs is related to the EV-associated miR-1246 expression *in vivo*, we implanted PANC-1 cells into the flanks of male nude mice and collected plasma samples at one- and two-weeks post implantation. EVs were isolated from the plasma and separated into the OP1 and OP2 group as described (Fig 1). Total RNA was extracted from the isolated EVs and expression of human specific miR-1246 was analyzed by qRT-PCR. As shown in Fig 4, miR-1246 expression in the OP1 group (Fig 4, top right), but not in the OP2 group (Fig 4, top left), seems to be higher in PANC-1 xenografted mice versus control mice, at two weeks post implantation. These results suggest the feasibility of our new simplified EV isolation protocol in clinical studies measuring miRNA levels in the OP1 fraction from PDAC patient plasma.

### miRNA expression in plasma EVs of PDAC patients

As a proof of principle, we collected 7 plasma samples from patients with localized PDAC (stage I to IIA; see Table 1) and 7 plasma samples from age- and sex-matched controls. Plasma EVs were isolated and separated into the OP1 and OP2 groups and verified by the nanoparticle tracking analysis (Fig 2A). Because plasma exosome miR-196a expression was previously found to be significantly elevated in PDAC patients [22], we analyzed miR-196a expression in the isolated EVs from the patient plasma samples. Consistent with our cell line study (Fig 3), miR-196a expression was significantly higher in the OP1 group, not the OP2 group, in PDAC patients as compared to healthy subjects (Fig 4, low panel). However, expression of miR-1246 was unchanged in plasma EVs derived from PDAC patients relative to control subjects, irrespective of EV sizes (Fig 4, low panel). While this is in contrast to our cell line-based results, it is in line with our recent report that plasma exosome miR-1246 levels are not elevated in patients with early stage PDAC [22].

## Discussion

Expression of exosome associated miRNAs has been explored as potential biomarkers for PDAC [25]. Yet reliable and sensitive exosome miRNA markers have not been established for this malignancy. This is in part due to the fact that EVs are derived from different cellular sources and present in various sizes [8] and that conventional methods of exosome isolation, namely ultracentrifugation and polymer-based commercial kits, give rise to a mixed population of EVs. miRNA expression among different groups of EVs could significantly differ, which compromises the sensitivity and specificity of circulating exosome miRNAs for PDAC detection. Whereas different molecular profiles in extracellular vesicles of different sizes have been described [26, 27], miRNA profiles among different groups of EVs derived from PDAC cells were previously uncharacterized. In the present study, we have developed a simple protocol, using centrifugation, sequential filtration and ultracentrifugation, to successfully separate EVs (traditionally considered exosomes) into different groups. We found that exosomes are enriched in the OP1 fraction as evidenced by positive and negative markers for exosomes, indicating a good separation of exosomes from other EVs. Furthermore, we demonstrated that miR-196a and miR-1246 expression significantly differs between OP1 and OP2 groups, implying that the OP1 group enriches biomarkers for PDAC. This is true for both PDAC cell line-derived EVs and those from xenograft nude mouse and PDAC patient plasma. Thus, we have achieved the isolation of a size-restricted uniform population of EVs enriched with exosomes and exosome miRNAs indicative of PDAC. The results from this study indicate that size-restricted isolation of plasma EVs is a new strategy in the development of plasma exosome miRNAs as biomarkers for PDAC.

Most previous studies of EV associated miRNA expression in cancer focuses on exosome miRNAs in biological fluids [22, 25, 28]. However, as mentioned earlier, the most commonly used methods for exosome isolation often result in a mixed EV population, consisting of exosomes, microvesicles, and potentially other protein complexes [8]. While disagreements over the exact size of exosomes remain [29–32], several lines of evidence from the present study support the idea that exosomes are a group of EVs at sizes of approximately 100nm and smaller. First, we were able to separate PDAC EVs into an OP1 group with an average diameter of 100nm or less, and an OP2 group with an average diameter ranging from 100-220nm, as verified by nanoparticle analysis. Second, the successful separation of the two groups of EVs provided us an opportunity to determine which group of EVs is enriched in exosomes. Our results show that exosome markers are primarily present in the OP1 group, not in the OP2 and OP3 groups, indicating that exosomes are highly enriched in the smaller EV group. The presence of OP2 was evident as shown by nanoparticle analysis, Coomassie blue staining, and protein and RNA measurements (S1 Table). However, it seems that miRNA expression levels are generally lower in this fraction of EVs (Fig 3A). The true nature of the OP2 group remains to be revealed. Third, these indications were further strengthened by our findings that expression of miR-1246 and miR-196a, two miRNAs highly enriched in PDAC cell line-derived exosomes [22], is significantly higher in the OP1 group compared with the OP2 and OP3 groups. Our findings thus reinforce the concept that exosomes are a subset of EVs with smaller diameters as compared with other EV groups, and that selectively enriched exosome miRNAs are potentially sensitive biomarkers for PDAC.

The limitations of this study include the small sample size of PDAC patient plasma, which does not allow an accurate estimation of the sensitivity and specificity of the miRNA signatures in size-restricted plasma exosomes versus those in other EV groups for PDAC detection; and a lack of a direct comparison of this new exosome isolation protocol with other commonly used methods in detecting PDAC by plasma exosome miRNA signatures, largely due to a limited volume of plasmas available for this study. Future studies are required to address these limitations.

## Conclusions

In summary, the present study demonstrated a simple protocol to separate exosome enriched small EVs from other EV groups derived from PDAC cells and human plasma, and the enhanced expression of the highly enriched PDAC exosome miRNAs in the isolated small EV group. Further studies are warranted to test this protocol in a cohort of PDAC patient plasma samples in order to develop exosome miRNAs as circulating biomarkers for early stage PDAC.

## Supporting information

S1 Raw images (PDF)

S1 Table Protein and RNA concentrations of OP1 and OP2 EVs derived from 20 mL culture medium of PANC-1 cells. (DOCX)

S1 File Minimal data set for Figs 3 and 4. (PDF)

## Abbreviations

EVs: extracellular vesicles
FBS: fetal bovine serum
IRB: Institutional Review Board
miRNA: microRNA
OUHSC: University of Oklahoma Health Sciences Center
PDAC: pancreatic ductal adenocarcinoma
RT-PCR: Reverse transcription polymerase chain reaction
