# Supplementary material for: Isolation of extra-cellular vesicles in the context of pancreatic adenocarcinomas: Addition of one stringent filtration step improves recovery of specific microRNAs
Source: PLoS One. 2021 Nov 16;16(11):e0259563. doi: 10.1371/journal.pone.0259563 (PMC8594802; doi:10.1371/journal.pone.0259563)

Loading Order for All Images: Loaded 1st→ → → Last

Image was used for
Figure 2.B CD63

Image was captured using the Licor Odyssey imaging system

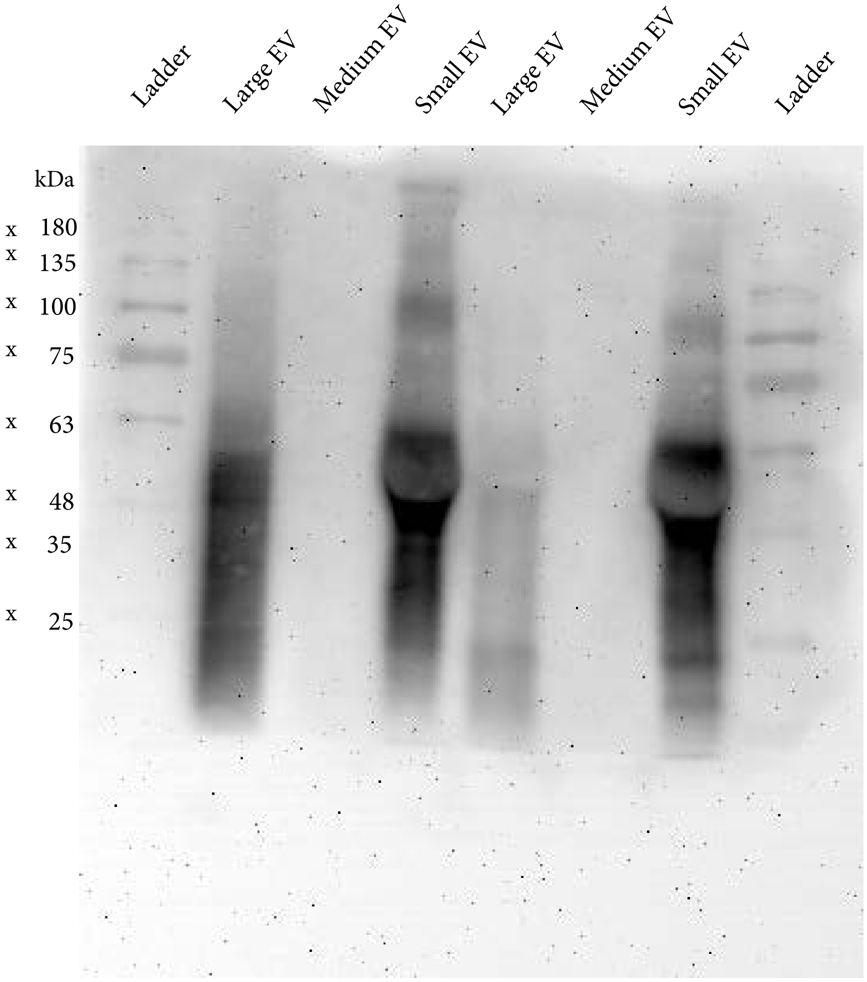

Image was used for
Figure 2.B CD81

Image was captured using the Licor Odyssey imaging system

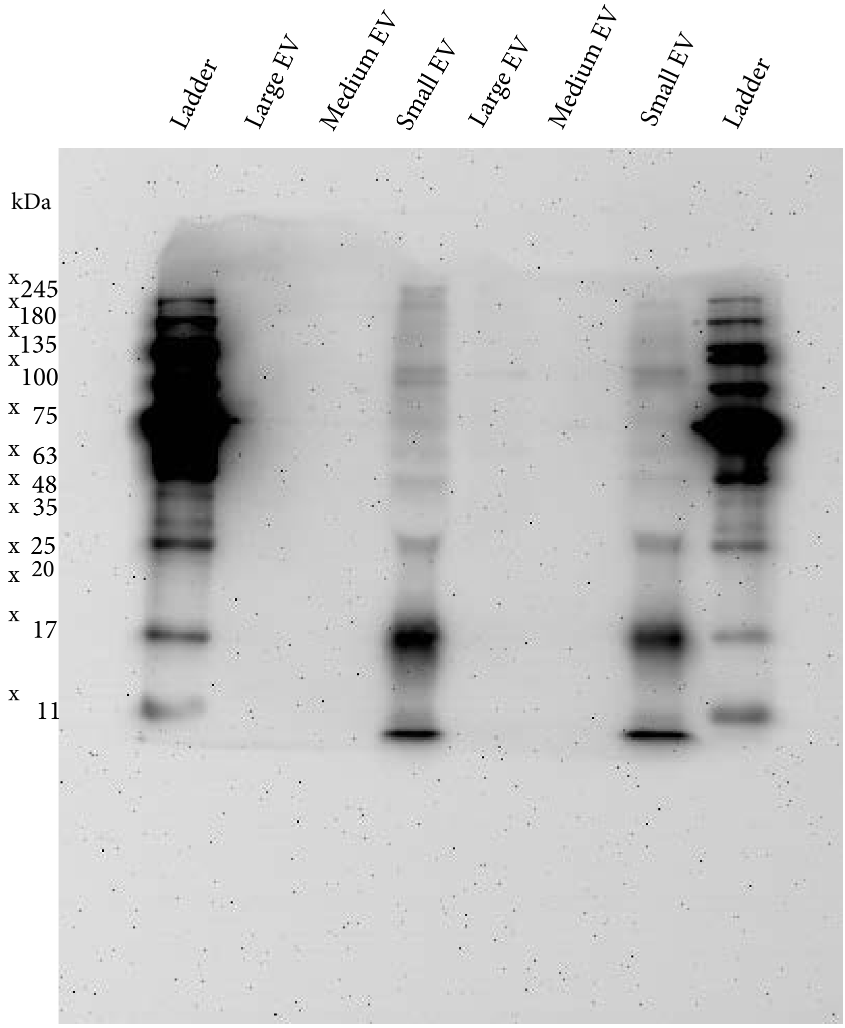

Image was used for
Figure 2.B TSG101

Image was captured using the Licor Odyssey imaging system

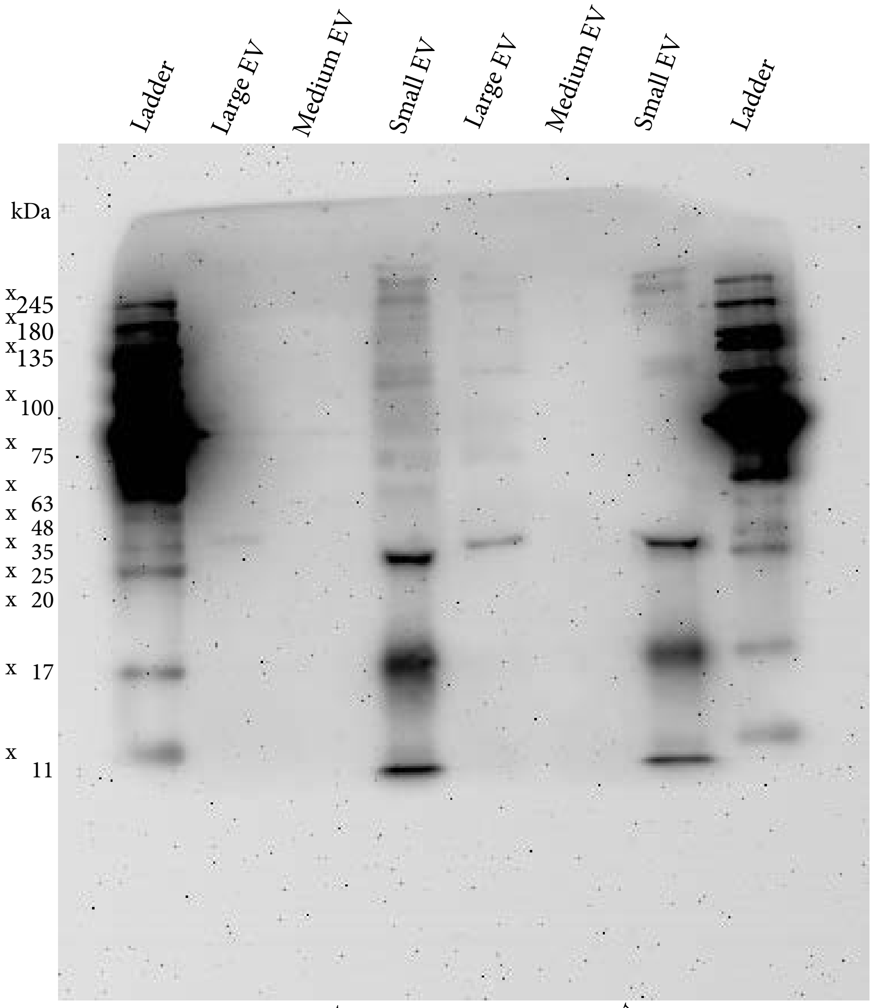

Image was used for
Figure 2.B Flotillin-1

Image was captured using the Licor Odyssey imaging system

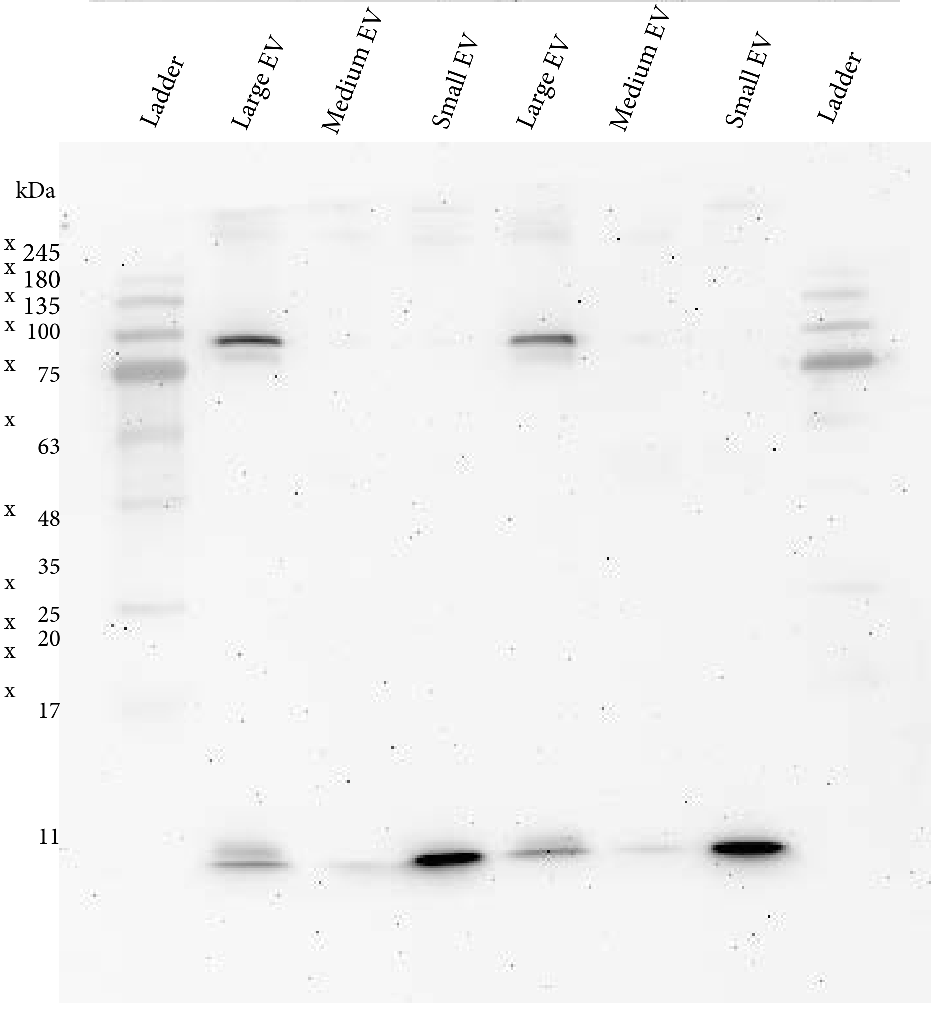

Image was used for Figure 2.B Calnexin

Image was captured using the Licor Odyssey imaging system

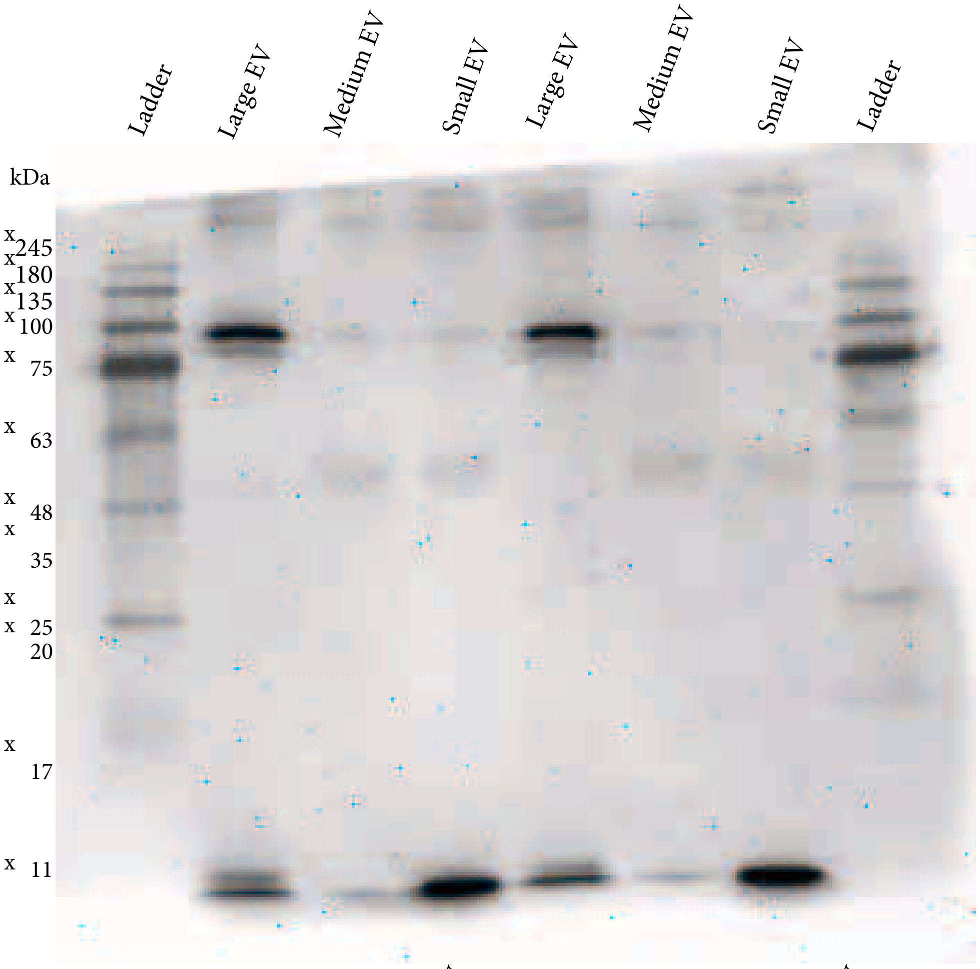

Image was used for Figure 2.C

Smart phone was used to capture this image.

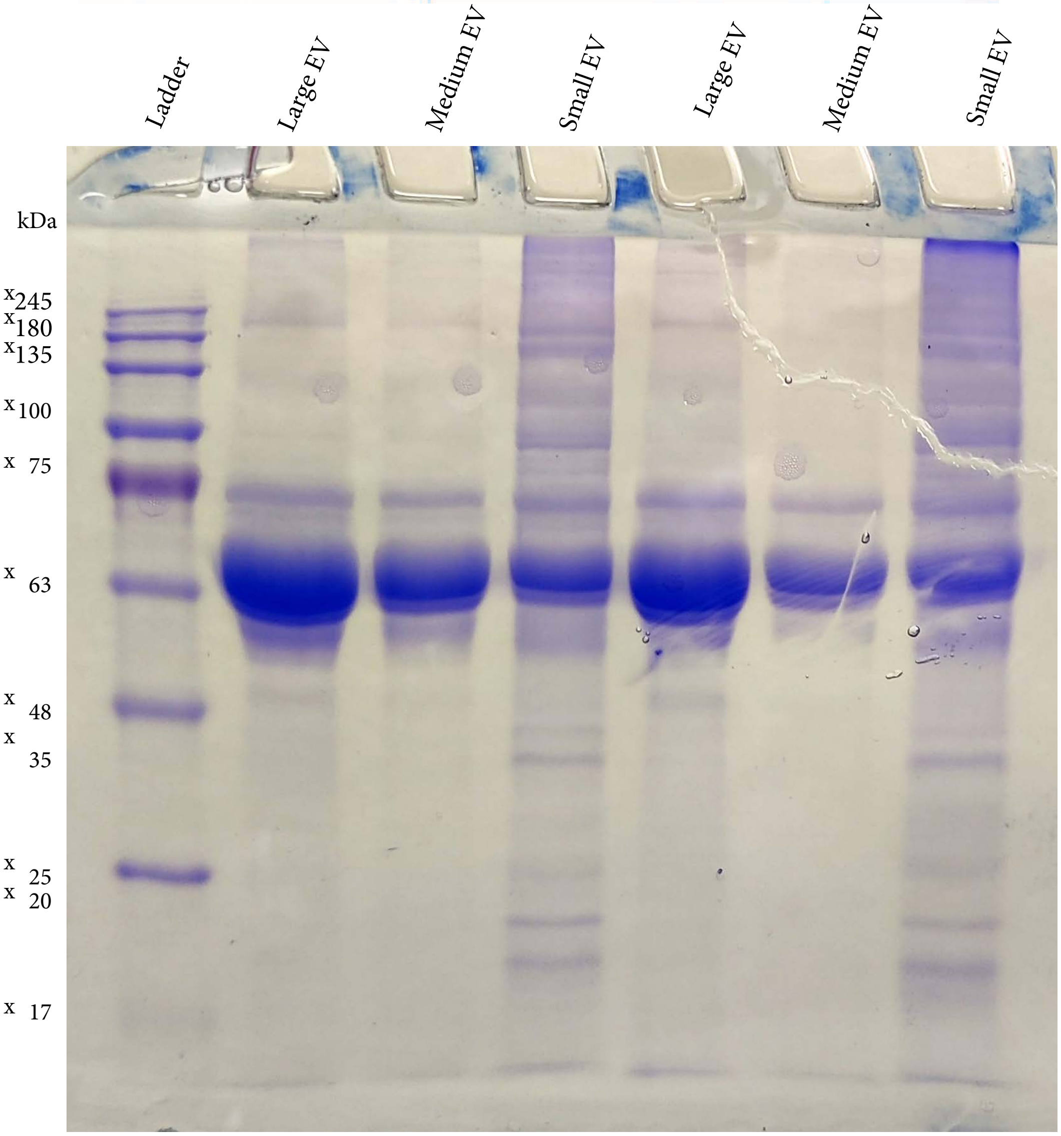

Supplement: S1 Raw images — (PDF) [file pone.0259563.s001.pdf]
