# Supplementary material for: Isolation of extra-cellular vesicles in the context of pancreatic adenocarcinomas: Addition of one stringent filtration step improves recovery of specific microRNAs
Source: PLoS One. 2021 Nov 16;16(11):e0259563. doi: 10.1371/journal.pone.0259563 (PMC8594802; doi:10.1371/journal.pone.0259563)
Supplement: S1 Table — (DOCX) [file pone.0259563.s002.docx]

|  | OP2 | OP1 |
| --- | --- | --- |
| Protein (µg) | 171.2 ± 49.64 | 331.8 ± 90.38 |
| RNA (ng) | 149.7 ± 22.71 | 226.7 ± 58.32 |

**Supplement Table 1. Protein and RNA concentrations of OP1 and OP2 EVs derived from 20 mL culture medium of PANC-1 cells.** Cells were 70% confluence when medium was exchanged with fresh DMEM. OP1 and OP2 EVs were isolated 24 h later, and proteins and RNAs were extracted. Protein concentration was determined by BCA assay, and RNA concentration by NanoDrop ND-100 Spectrophotometer (n=3-6, mean ± SEM).

Minimal dataset

| OP2 | OP1 |
| --- | --- |
| 394.2420 | 543.1410 |
| 232.1525 | 678.1554 |
| 94.4443 | 217.7911 |
| 116.1025 | 164.1400 |
| 96.0498 | 169.6082 |
| 94.4443 | 217.7911 |

| OP2 | OP1 |
| --- | --- |
| 124.5 | 132.0 |
| 195.0 | 215.0 |
| 129.5 | 333.0 |

Protein (µg) RNA (ng)
