# Supplementary material for: Isolation of extra-cellular vesicles in the context of pancreatic adenocarcinomas: Addition of one stringent filtration step improves recovery of specific microRNAs
Source: PLoS One. 2021 Nov 16;16(11):e0259563. doi: 10.1371/journal.pone.0259563 (PMC8594802; doi:10.1371/journal.pone.0259563)
Supplement: S1 File — (PDF) [file pone.0259563.s003.pdf]

Suppl_data for Fig3 A. Relative miRNA levels, based on the $\Delta\Delta C_T$ method.

miRNAs	OP3	OP2	OP1
miR-1246	1.819873	1.3	6.93461
	1.305422	0.7	6.052264
	1.819873	1	6.93461
miR-196a	7.295095	1.2	11.19928
	8.568819	1	13.76176
	7.295095	0.8	11.19928
miR-3605	12.564983	0.8	1.488867
	18.821156	1	2.841991
	19.218442	1.2	7.541803
miR-21	202.736144	1.2	41.670766
	122.012663	1	28.449703
	111.457192	0.8	27.935418

Suppl_data for Fig3 B. Relative miRNA levels, based on the $\Delta\Delta C_T$ method.

miRNA levels (fold change)	HPNE	PANC-1
miR-1246 OP2	1	6.723984
	0.8	6.786023
	1.2	3.669651
	1	3.476510
miR-1246 OP1	0.7	34.14253
	1.3	55.44912
	0.8	26.71135
	1.2	30.66402
miR-196a OP2	0.8	11.84539
	1.2	22.34122
	0.9	2.145134
	1.1	2.875453
miR-196a OP1	0.65	12.09881
	1.35	21.57856
	0.95	80.34367
	1.15	88.51638

Suppl_data for Fig4 top panel. Relative miR-1246 levels, based on the $\Delta\Delta C_T$ method.

miR-1246 levels	W0	W1	W2
OP2	1	3.63291	1.61798
	0.9633583	0.4190783	0.3693524
	0.6758769	1.795323	0.835811
	1.409444	0.4900596	0.5764639
	0.2247809	0.7094151	1.081884
	1.72654	0.4036438	0.8731579
OP1	2.031283	0.8383248	13.80263
	0.564583	0.2064867	1.724645
	0.447803	0.6530507	4.72873
	1.09115	1.337903	0.4518902
	1.076826	0.8418671	2.149487
	0.788357	1.463736	0.2405237

Suppl_data for Fig4 middle panel. Tumor volume and mouse weight at week 0, 1 and 2

Tumor volume (mm^3)	W0	W1	W2
	0	21.904	103.192
	0	18.513	38.4
	0	35.378	56.25
	0	38.475	50.625
	0	42.336	55
	0	46.08	107.909

Mouse weight (g)	W0	W1	W2
	19	21.9	22.3
	22.8	20.8	22.4
	24.36	24.3	21.1
	19.8	25.7	26.8
	20.4	20.7	25.6
	22	21.5	21.2

Suppl_data for Fig4 low panel. Relative miRNA levels, based on the $\Delta\Delta C_T$ method.

miR-1246 levels

plasma OP2		plasma OP1	
Control	PDAC	Control	PDAC
0.727728	1.759986	0.844708	0.408801
0.249403	1.106365	0.360106	0.299889
0.600598	0.735868	1.294382	1.339754
0.674377	1.903366	2.209409	0.659504
1.620727	2.205205	0.652844	0.266236
1.729506	1.032097	0.504744	1.520524
1.397659	1.012837	1.133811	0.25058

miR-196a levels

plasma OP2		plasma OP1	
Control	PDAC	Control	PDAC
1.000161	0.809596	0.946669	1.517424
0.533191	1.634383	0.946669	2.64597
0.642765	0.509645	0.951804	0.946669
0.5054	1.642208	0.951804	2.792561
1.448066	1.190745	0.857037	1.106981
0.774407	0.678872	1.175067	2.201781
2.096013	0.825353	1.170952	0.985974
